# MeganServer: facilitating interactive access to metagenomic data on a server

**DOI:** 10.1093/bioinformatics/btad105

**Published:** 2023-02-24

**Authors:** Anupam Gautam, Wenhuan Zeng, Daniel H Huson

**Affiliations:** Algorithms in Bioinformatics, Institute for Bioinformatics and Medical Informatics, University of Tübingen, Tübingen 72076, Germany; International Max Planck Research School “From Molecules to Organisms”, Max Planck Institute for Biology Tübingen, Tübingen 72076, Germany; Cluster of Excellence: EXC 2124: Controlling Microbes to Fight Infection, University of Tübingen, Tübingen 72076, Germany; Algorithms in Bioinformatics, Institute for Bioinformatics and Medical Informatics, University of Tübingen, Tübingen 72076, Germany; Algorithms in Bioinformatics, Institute for Bioinformatics and Medical Informatics, University of Tübingen, Tübingen 72076, Germany; International Max Planck Research School “From Molecules to Organisms”, Max Planck Institute for Biology Tübingen, Tübingen 72076, Germany; Cluster of Excellence: EXC 2124: Controlling Microbes to Fight Infection, University of Tübingen, Tübingen 72076, Germany

## Abstract

**Motivation:**

Metagenomic projects often involve large numbers of large sequencing datasets (totaling hundreds of gigabytes of data). Thus, computational preprocessing and analysis are usually performed on a server. The results of such analyses are then usually explored interactively. One approach is to use MEGAN, an interactive program that allows analysis and comparison of metagenomic datasets. Previous releases have required that the user first download the computed data from the server, an increasingly time-consuming process. Here, we present MeganServer, a stand-alone program that serves MEGAN files to the web, using a RESTful API, facilitating interactive analysis in MEGAN, without requiring prior download of the data. We describe a number of different application scenarios.

**Availability and implementation:**

MeganServer is provided as a stand-alone program tools/megan-server in the MEGAN software suite, available at https://software-ab.cs.uni-tuebingen.de/download/megan6. Source is available at: https://github.com/husonlab/megan-ce/tree/master/src/megan/ms.

**Supplementary information:**

Supplementary data are available at *Bioinformatics* online.

## 1 MEGAN

Metagenomics is the study of microbiomes using DNA sequencing. As the throughput and cost-efficiency of sequencing technologies continue to decrease, the number and size of metagenomic samples collected in a project continues to increase.

The first three basic computations performed on such data (after quality control), are taxonomic analysis, functional analysis and comparative analysis. One major approach is based on sequence alignment (Huson *et al.*, 2016), which is computationally intensive and thus usually performed on a server or ‘in the cloud’.

Here we focus on the analysis of metagenomic data using the DIAMOND + MEGAN pipeline (Bağcı *et al.*, 2021). This first employs the high-throughput alignment tool DIAMOND (Buchfink *et al.*, 2015) to align reads against a protein reference database such as NCBI-nr or AnnoTree (Gautam *et al.*, 2022). This gives rise to alignments in DAA (DIAMOND alignment archive) format. The second step is to perform taxonomic and functional binning of reads using MEGAN or the associated command-line tool daa-meganizer. This process extends the content of the DAA files to include classification information, resulting in ‘meganized’ DAA files. MEGAN (‘metagenome analyzer’) is an interactive application that runs on a personal computer. It comes with a number of command-line tools for analyzing data on a server. Meganized DAA files can be interactively explored and analyzed in MEGAN, and multiple files can be opened together in a single ‘comparison document’. MEGAN provides numerous features for interactive exploration, such as hierarchical displays of the NCBI (Federhen, 2012) and GTDB (Parks *et al.*, 2018) taxonomies and functional classifications, several types of charts, clustering algorithms, dialogs for accessing individual reads and alignments and gene-centric assembly.

## 2 MeganServer

One drawback of MEGAN has been that files must be located on the local machine to be opened. This required that users download files from the server to their personal computer. This is becoming increasing challenging, as the size of metagenomic projects continues to grow, and it discourages collaborators from ‘quickly taking a look’ at the data. To address this, here we present MeganServer, a light-weight, stand-alone web server that serves meganized DAA files, and other MEGAN-associated files, to the web, using a RESTful API, see also (Mitchell *et al.*, 2020; Paczian *et al.*, 2019). During setup of the server, a root directory is specified and then all appropriate files found in or below the root directory are served. The API provides endpoints for obtaining file-related information, classification-related information, for accessing reads and matches and for administrating the server.

**Fig. 1.**
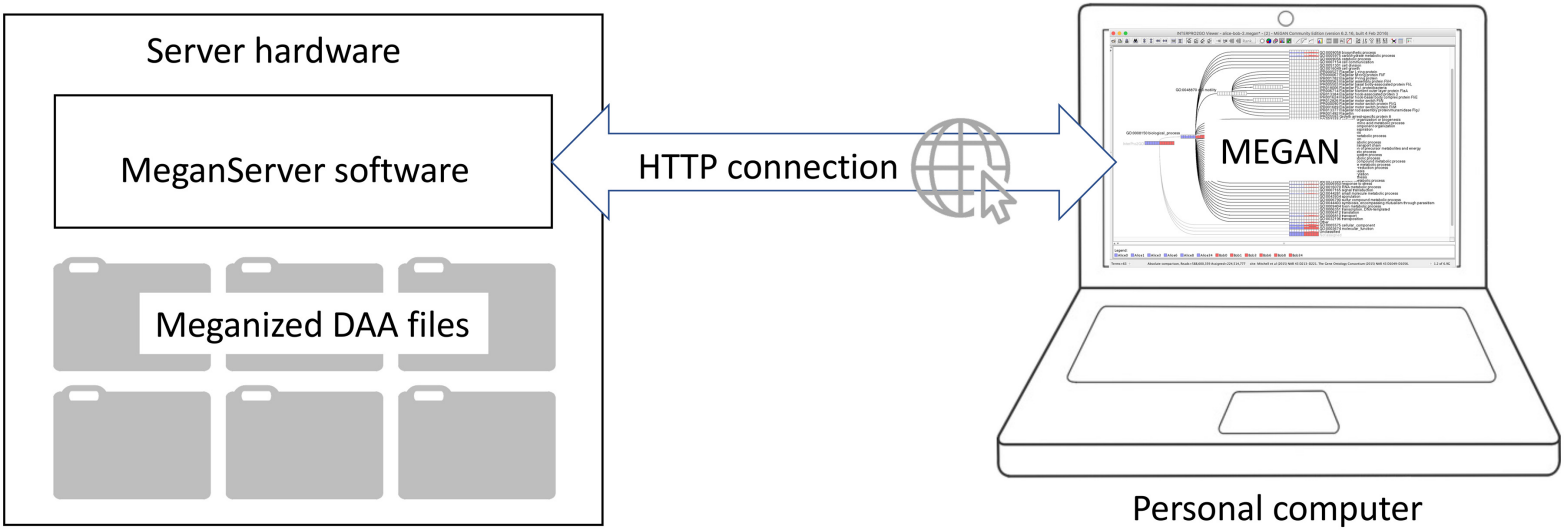
The MeganServer program runs on a server and makes meganized DAA files accessible to instances of MEGAN running on (remote) personal computers, via the web

The service can be accessed using a web browser. For example, the URL http://maira.cs.uni-tuebingen.de:8001/megan6server/help will display a help window. However, we have implemented client code in MEGAN and the program allows the user to interactively connect to a MeganServer instance (see Fig. 1). MEGAN displays a structured list of all files accessible from the server. Such files can be interactively opened and analyzed as if they were present on the local machine.

MeganServer is provided in the tools directory of the MEGAN installation. To launch the server, execute the following command:


megan-server -g -i 〈dir〉  -e 〈name〉  -p 〈port〉

Here, the option -g turns on guest access, 〈dir〉 is the directory containing all files to be served (including all directories below), 〈name〉 is the endpoint name, e.g. megan6server and 〈port〉 is the number of the port to be used, e.g. 8001. The program will run in the background until terminated. Execute the program with option -h to obtain a full list of options. See the online Supplementary Material for more details.

## 3 Applications

Here we illustrate the use of MeganServer using four examples.

### 3.1 Public MeganServer instance

We maintain a public instance of MeganServer reachable at http://maira.cs.uni-tuebingen.de:8001/megan6server/help, accessible with user-id guest and password guest. We use this instance for teaching purposes. It hosts several short-read datasets, including some kitten gut microbiomes, and over 80-long-read metagenomic datasets from several recently published papers.

### 3.2 International collaboration using MeganServer

Together with colleagues in Brazil, we performed short-read metagenomic analysis of six fruit-waste samples to study their potential bio-surfactant bio-synthesis (Silva *et al.*, 2022). Initial metagenomic analysis was performed on a server hosted in Tübingen, Germany, and datasets were then accessed from Sorocaba, Brazil, using MeganServer. It takes around 75 s to open all six samples in MEGAN via MeganServer.

### 3.3 MeganServer in a cloud environment

Computational analysis of metagenomic data is often performed ‘in the cloud’. The ‘de.NBI Cloud’ (German Network for Bioinformatics Infrastructure) provides configurable virtual machines (VM) for individual projects.

In one of our projects on this platform, we used a VM to run the DIAMOND + MEGAN pipeline on 172 short-read samples. To access to results, we setup an instance of MeganServer on the VM. In the following, 〈dir〉 represents the path to the directory to be served. Access to the service requires ssh tunneling using an access key, 〈key〉, user name, 〈user〉 and the IP address of the VM, 〈IP〉, as follows:


ssh -i 〈key〉 \\ -L 127.0.0.1:8080:localhost:8080 〈user〉 @ 〈IP〉

Then MeganServer is launched as follows (here 〈name〉 is the desired endpoint name):


megan-server -g -i 〈dir〉  -e 〈name〉  -p 8080

Opening all 172 samples simultaneously in MEGAN took 10 min.

### 3.4 Very large projects

In a current project, we are analyzing 687 short-read metagenomic datasets, consisting of 4.4 TB of data in total. These data are hosted on a local linux server and we use MeganServer to access it. Loading all 687 samples into MEGAN on a laptop via MeganServer took under 2 h. This loaded all classifications for all samples and most interactive analyses do not require additional contact with the server, unless the user wants to view alignments or download reads.

## 4 Discussion

Metagenome datasets are usually analyzed on servers and are accessed via web-interfaces or downloaded and analyzed using scripts or interactive programs. MeganServer allows researchers to analyze large datasets using the interactive program MEGAN, without the need to download them.

## Supplementary Material

btad105_Supplementary_Data
