# Supplementary material for: MeganServer: facilitating interactive access to metagenomic data on a server
Source: Bioinformatics. 2023 Feb 24;39(3):btad105. doi: 10.1093/bioinformatics/btad105 (PMC9991050; doi:10.1093/bioinformatics/btad105)
Supplement: btad105_Supplementary_Data [file btad105_supplementary_data.pdf]

Supplementary material for "MeganServer: facilitating interactive access to metagenomic data on a server"

A. Gautam, W. Zeng and D.H. Huson

Here we present an example on how to run MeganServer using data from our recently published work on fruit-waste fermentation metagenomic samples (Silva *et al.*, 2022). In this study, MeganServer was used to provide access to the data for our collaborators in Brazil.

1 Starting a MeganServer instance

The directory to be served, `fiori2022`, contains six DAA files (Supplementary Figure S1), which have already been processed by the DIAMOND+MEGAN pipeline. They represent fruit-waste fermentation samples (Silva *et al.*, 2022). The directory is hosted on an in-house server named `osa3` and MEGAN was installed on the server.

An instance of MeganServer is started by typing the following command:

`tools/megan-server -g true -i fiori2022 -e megan6server -p 8001`.

Here, the option `-g true` turns on "guest access" with user-id "guest" and password "guest". The directory to be served (option `-i`) is `fiori2022` and the endpoint name (option `-e`) is set to `megan6server`. The option `-i 8001` sets the port to be used for serving.

As shown in Suppplementary Figure S1a, the software lists the address at which the server can be reached, in this example, the URL is `http://osa3:8001/megan6server`. Please do not attempt to access this instance of MeganServer, as it is used here only for illustration purposes. A public instance of MeganServer can be accessed here: `http://maira.cs.uni-tuebingen.de:8001/megan6server/help`.

Note that when you run the software for the first time, then you will be prompted to provide a password for the username `admin`, and the program will generate a `.MeganServerUser.def` file in your home directory, which is used to maintain user data.

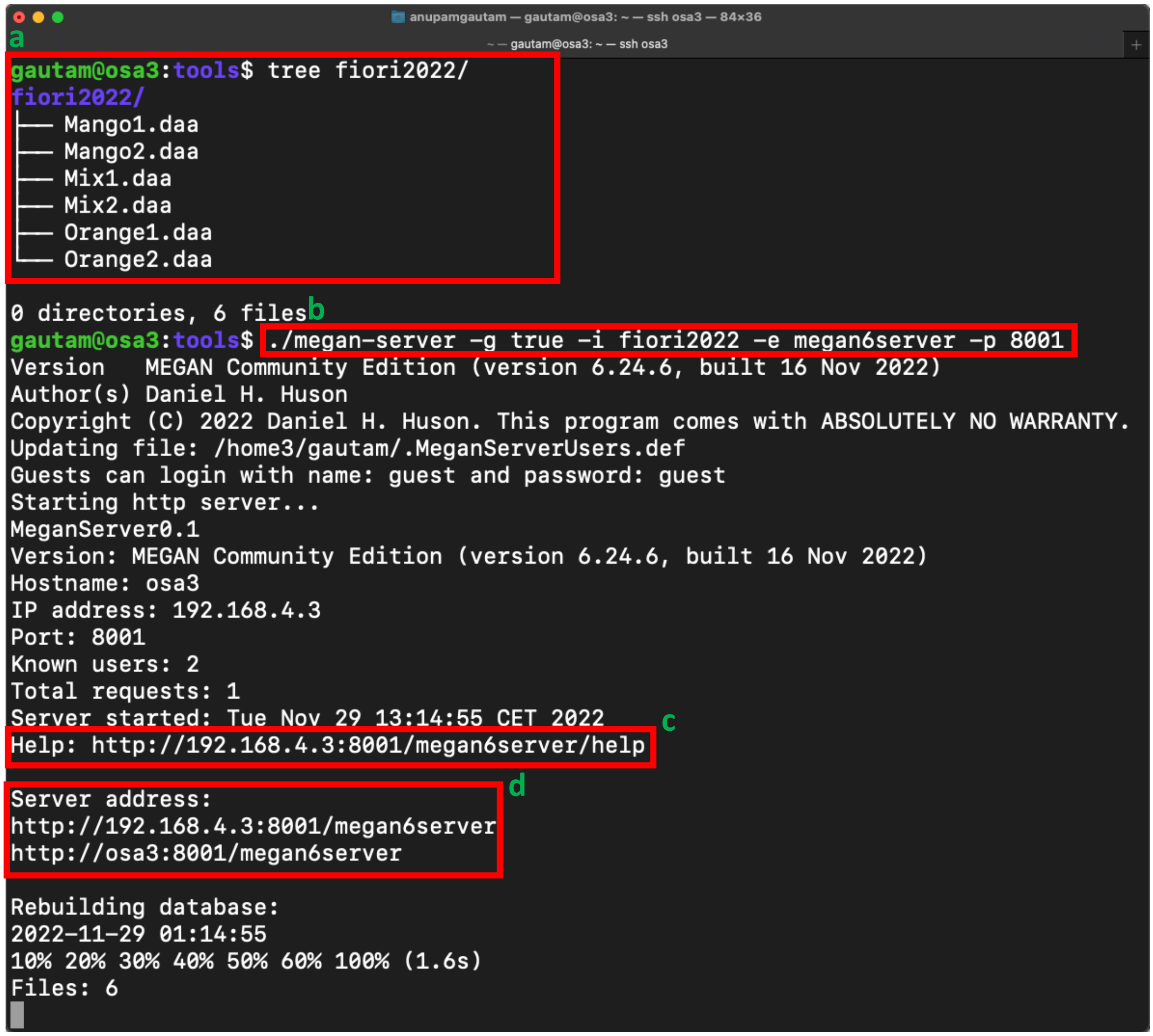

Figure S1: **Starting a MeganServer instance on server:** (a) In this example, the directory to be served is called `fiori2022` and is located on a computer called `osa3`. (b) This command launches MeganServer and the program will serve the files to the internet via the specified port. (c) Use URL into a web browser to obtain a help screen. (d) This base URL can be used in MEGAN to contact the server.

2 Accessing the served data from your personal computer

2.1 Web browser access

When MeganServer is launched, it lists an address (Supplementary Figure S1c) that can be used to access the service from a web browser (Supplementary Figure S2a), in this example, it is `http://osa3:8001/megan6server`. (This is an example URL, it is not externally reachable.) Specifying the resource `help` will display a help window (Supplementary Figure S2a). You can specify other resources listed on the help page to interact with your data. For example, specify the resource `list`, like this `http://osa3:8001/megan6server/list`, to obtain a list of all samples present in the served di-

rectory. The first time you access a resource other than **help**, you will be prompted for a user-id and password, such as `guest` and `guest` (Supplementary Figure S2b). In this example, we will obtain a list of the six files contained in the `fiori2022` directory (Supplementary Figure S2c). More information about MeganServer API commands with examples can be found in the detailed manual present at `http://software-ab.cs.uni-tuebingen.de/download/megan6/User_Manual_for_MeganServer.pdf`.

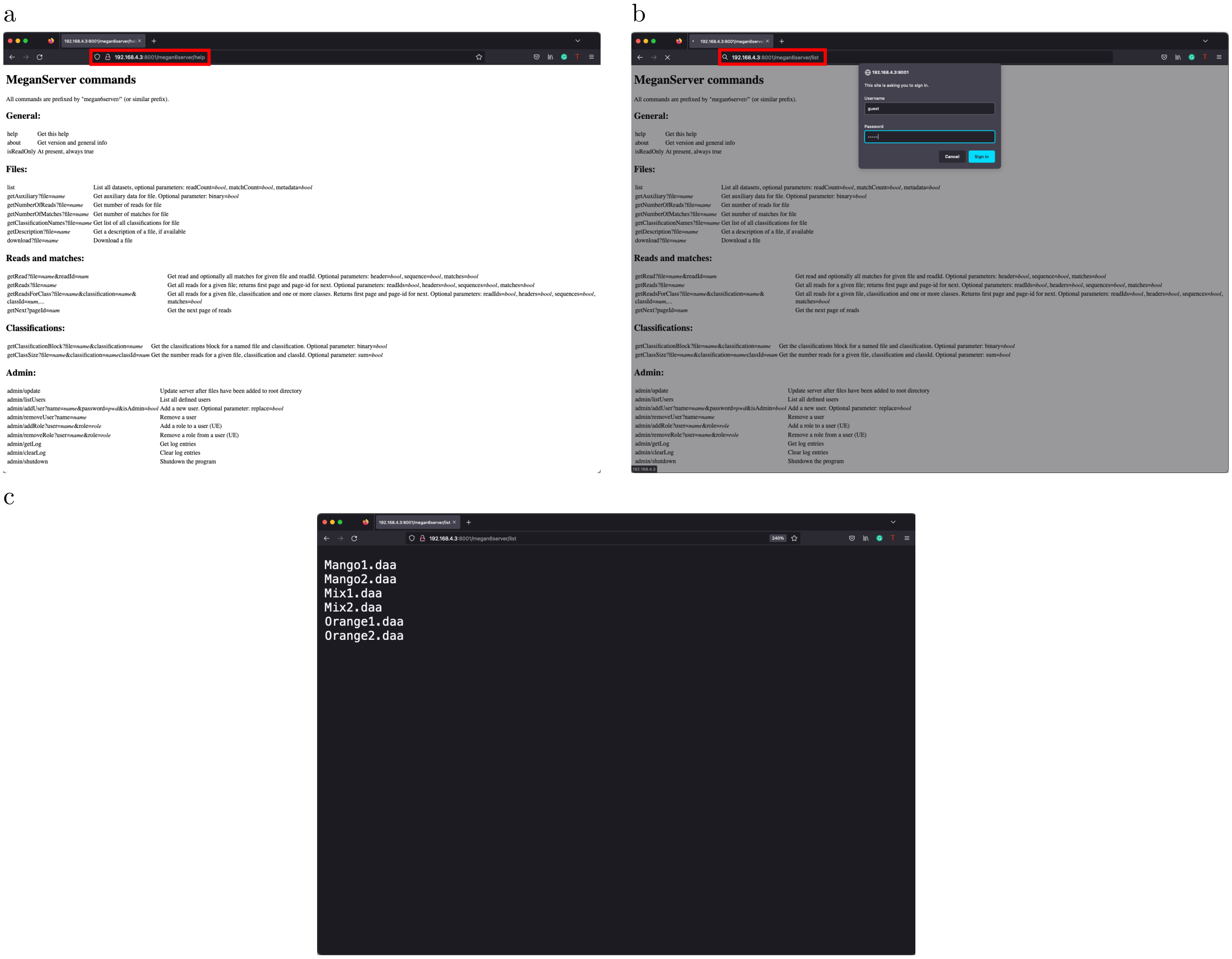

Figure S2: **Accessing and interacting with data through the web:** (a) Enter the MeganServer URL and a resource request into a web browser. For example, `http://osa3:8001/megan6server/help` requests the resource **help**, which results in the displaying of a help text. (b) Requesting the `list` resource, by typing `http://osa3:8001/megan6server/list`, results in a list of all served files (c). This may trigger an `authentication` dialog.

2.2 MEGAN access to served files

Files can also be accessed using the program MEGAN, which acts as a client for the server. To contact an instance of MeganServer, select the `File` → `Open From Server...` menu item (Supplementary Figure S3a). In the dialog window (Supplementary Figure S3b), enter the server endpoint and user credentials (for example, user `guest` and password `guest`).

The program then displays a list of all available remote files (Supplementary Figure S3c). Select and open

any files of interest. Multiple files can also be opened together in a single document using the `Compare` button (Supplementary Figure S3d). Then, clicking the `Apply` button will show the selected file or files in the default NCBI taxonomy viewer (Supplementary Figure S3e). Further one can view alignments (Supplementary Figure S4d), inspect reads (Supplementary Figure S4e) by opening any file or files of interest. For more details, see (Bağcı *et al.*, 2021) or the MEGAN manual at `http://software-ab.cs.uni-tuebingen.de/download/megan6/manual.pdf`

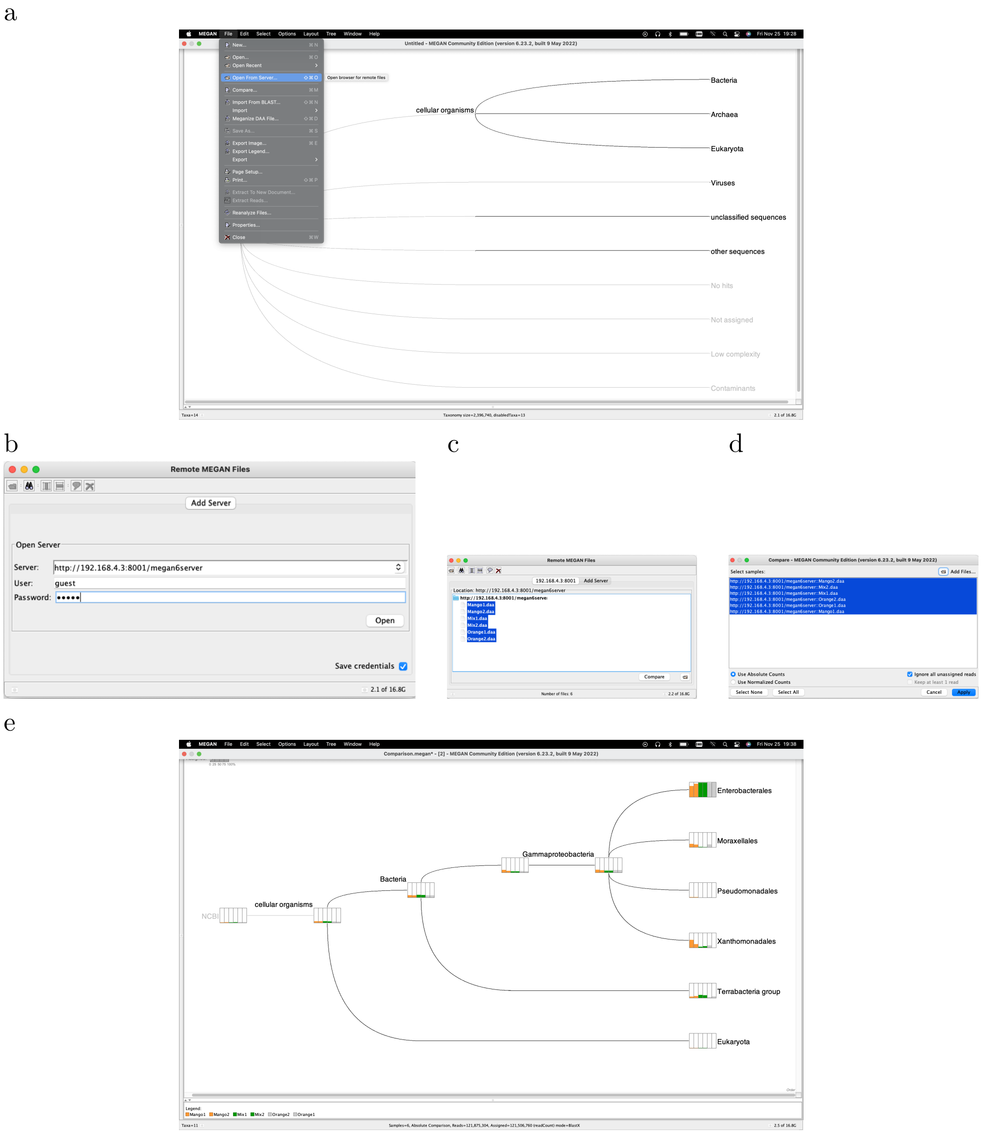

Figure S3: **Accessing and interacting via MEGAN-GUI:** (a) To access an instance of MeganServer, select the `File` → `Open From Server...` menu item and then (b) enter the server endpoint and user credentials. (c) After successfully contacting the service, an overview of all available remote files is displayed. (d) If more than one file is selected, then one can use the `Compare` dialog to combine multiple files into a single document. (e) Pressing `Apply` will open the files in the default NCBI taxonomy viewer.

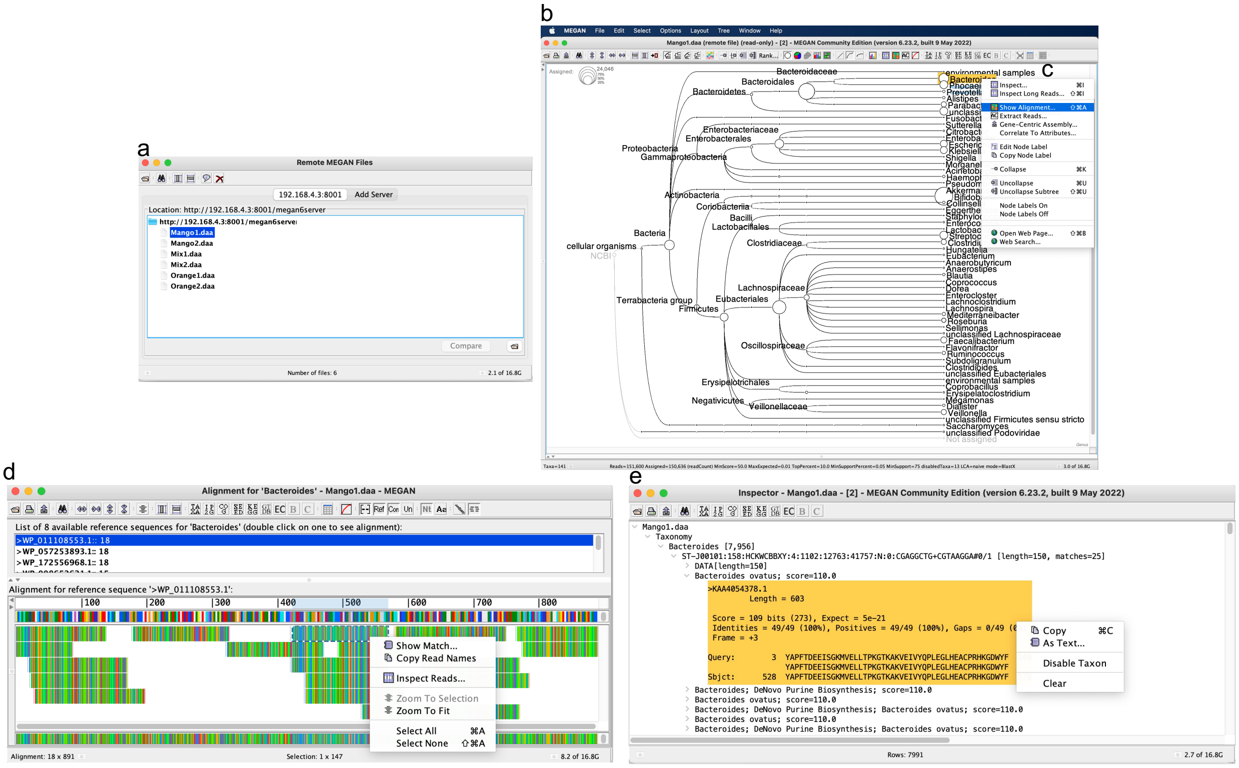

Figure S4: **Accessing and interacting via MEGAN-GUI continued:** (a) Double-click a sample of interest from the list of available remote files. (b) This will open the file in the default NCBI taxonomy viewer. (c) Select a node or leaf of interest. This will display the number of reads assigned and summed to that node or leaf. Right-click on a node to obtain a context menu, showing various analysis options. (d) The `Show Alignment...` menu item opens the Alignment dialog for the corresponding node. (e) The `Inspect...` menu item opens the inspector window for the corresponding node.

References

Bağcı, C., Patz, S., and Huson, D. H. (2021). DIAMOND+ MEGAN: fast and easy taxonomic and functional analysis of short and long microbiome sequences. *Current protocols*, **1**(3), e59.

Silva, G. F. d., Gautam, A., Duarte, I. C. S., Delforno, T. P., Oliveira, V. M. d., and Huson, D. H. (2022). Interactive analysis of biosurfactants in fruit-waste fermentation samples using BioSurfDB and MEGAN. *Scientific reports*, **12**(1), 1–11.
